# Targeted Gene Delivery: Where to Land

**DOI:** 10.3389/fgeed.2020.609650

**Published:** 2021-01-20

**Authors:** Giulia Pavani, Mario Amendola

**Affiliations:** INTEGRARE, UMR_S951, Genethon, Inserm, Univ Evry, Univ Paris-Saclay, Evry, France

**Keywords:** genome editing, gene therapy, nuclease, CRISPR, targeted integration (TI), knock-in, safe harbor, homologous recombination (HR)

## Abstract

Genome-editing technologies have the potential to correct most genetic defects involved in blood disorders. In contrast to mutation-specific editing, targeted gene insertion can correct most of the mutations affecting the same gene with a single therapeutic strategy (gene replacement) or provide novel functions to edited cells (gene addition). Targeting a selected genomic harbor can reduce insertional mutagenesis risk, while enabling the exploitation of endogenous promoters, or selected chromatin contexts, to achieve specific transgene expression levels/patterns and the modulation of disease-modifier genes. In this review, we will discuss targeted gene insertion and the advantages and limitations of different genomic harbors currently under investigation for various gene therapy applications.

## Introduction

Blood genetic disorders are caused by mutations in genes or in their regulatory elements that result in a dysfunctional, dysregulated, or absent protein. Conventional gene therapy approach consists of the addition of a functional copy of a mutated gene to patients' cells using viral vectors, such as adeno-associated virus (AAV) (Mingozzi and High, 2011) and lentivirus (LV)-derived vectors (Naldini, 2011). These modified viruses can deliver the transgene expression cassettes encoded in their genome to the cell nucleus, where the genetic information is used. This gene replacement strategy is mutation-independent and thus can benefit patients with the same condition regardless of their genotype.

Despite its remarkable success for *ex vivo* and *in vivo* treatment of several monogenic disorders (Dunbar et al., 2018), there are still major hurdles to overcome to improve therapeutic outcomes and treat challenging monogenic (e.g., hemoglobinopathies, immunodeficiencies, and congenital anemias) as well as multifactorial blood diseases (e.g., cancer, autoimmune, and infectious disorders). Apart from vector-specific issues such as immunogenicity and tropism (Masat et al., 2013; Colella et al., 2018), which are beyond the scope of this review, classic gene replacement has a major limitation: it is hard to faithfully re-create characteristics of endogenous promoters and gene-specific regulation within the context of a viral vector. Tissue-, developmental-, and stimulus-specific gene expression requires the complex interaction of different genomic elements (promoters, enhancers, and silencers) that can be located in distant regions of the genome and span several kilobases (Schoenfelder and Fraser, 2019).

AAV vectors are small viruses (~4.7 kb), limiting the choice of regulatory elements to include in the expression cassette, especially when delivering large transgenes (Li and Samulski, 2020). Moreover, they persist mainly as episomes in non-dividing cells and are progressively lost through cell division (Nakai et al., 2001; Ehrhardt et al., 2003; Bortolussi et al., 2014)—a major obstacle for treating infantile disorders and tissues undergoing rapid proliferation (e.g., hematopoietic and epithelial cells). On the other hand, LV have larger cargo capacity (~8 kb), stably integrate in the genome, and persist through cell replication (Naldini et al., 1996), but they carry the intrinsic risk of insertional mutagenesis and oncogene transactivation (mainly when strong promoters/enhancers are present (Cavazzana et al., 2019; Bushman, 2020)). In addition, their semi-random integration (Schroder et al., 2002) results in transduction mosaicism and heterogeneous transgene expression due to chromatin position effects (Chen et al., 2017; Vansant et al., 2020), making therapeutic levels harder to reach.

When combining AAV and nucleases, both transgene expression cassettes and genomic integration sites contribute to the corrective strategy, dramatically expanding therapeutic possibilities. Primarily, targeting a functional copy of a gene to its endogenous locus, under the control of its own promoter and in the right chromatin context, can result in physiological expression and minimize genotoxic integrations. Alternatively, transgenes can be targeted to safe integration sites or specific genomic elements of interest to engineer cells with novel functions, further improving safety and increasing potential applications of gene replacement/addition therapy (Cox et al., 2015).

Sequence-specific endonucleases (such as ZNF, TALEN, or CRISPR/Cas9) (Gaj et al., 2016) can induce genomic DNA double-strand breaks (DSB) in proximity to pathological mutations and activate cellular DNA repair pathways to correct them. The inclusion of short single-stranded oligodeoxynucleotide (ssODN) donors is a simple and effective approach for precise correction of single-nucleotide mutations (DeWitt et al., 2016; De Ravin et al., 2017; Romero et al., 2019). Although their short size currently limits their application for diseases caused by multiple pathological variants (e.g., β-thalassemia, ~300 different mutations across the β-globin locus), technological advances in long ssODN synthesis would most likely expand their therapeutic potential (Praetorius et al., 2017; Roth et al., 2018).

DSB generated by endonucleases can also facilitate integration of therapeutic transgenes to selected genomic locations (targeted gene replacement). AAV has a tendency to integrate at pre-existing chromosomal breaks that provide free DNA ends for non-homologous end joining (NHEJ) (Miller et al., 2004). To increase efficiency, specificity, and precision of integration, homology arms derived from genomic regions flanking the target site are introduced on each side of the AAV cassette with the aim of leveraging the homologous DNA repair pathway (Hirata et al., 2002). Although effective in proliferating cells, homologous recombination is quite inefficient in quiescent hematopoietic stem cells (HSC) and postmitotic cells or tissues (Nishiyama, 2019; Shin et al., 2020). Therefore, alternative DNA repair mechanisms based on NHEJ or microhomology-mediated end joining (MMEJ) are now being investigated (Suzuki et al., 2016; Banan, 2020). In both cases, AAV are the gold-standard DNA delivery system for gene-targeted integration *in vivo* (Li et al., 2011) and *ex vivo* (Wang et al., 2015), though the exact molecular mechanism underpinning this process remains unknown (Deyle and Russell, 2009).

## Integration Strategies

Selecting a suitable genomic site for transgene integration depends on many factors, such as the expression level required, the target cells/tissue, and the disease to be treated.

We have subdivided integration sites in four groups according to functional characteristics: (i) endogenous promoters, when promoterless transgenes are inserted under the control of endogenous enhancers/promoters; (ii) safe genomic harbors, when transgenes and their promoters are integrated into genomic regions that allow robust expression without affecting cell physiology; (iii) disease modifier genes, when transgenes integrate into coding sequence of endogenous genes, whose inactivation benefits disease-affected cells; and (iv) specificity exchange, when transgenes are integrated into coding sequence of endogenous genes to change their function.

It is worth noting that this subdivision is only a working framework, as the same integration site can fall into two or more categories, and it is not exhaustive, as new integration strategies are described every day.

## Endogenous Promoters

### Correction of Dysfunctional Genes

A straightforward approach for targeted gene replacement consists in inserting a functional copy of a gene downstream of its endogenous promoter. This strategy can correct most pathological mutations that are scattered along the gene body (such as substitutions and frameshift mutations), while maintaining physiological gene expression (Table 1A), which can be hard to achieve with artificial promoters used in classical gene therapy vectors (Toscano et al., 2011).

**Table 1:** **(A–F)** The advantages and disadvantages of different integration strategies.

	**Integration strategies**		**Advantages**	**Disadvantages**	**References**
A	Endogenous locus	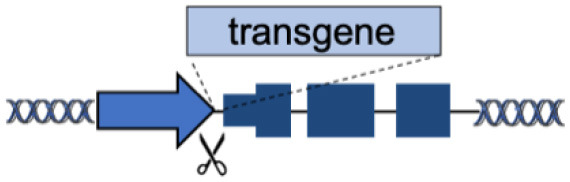	Physiological transgene expression Corrects multiple mutations	Gene-specific strategy Limited to gene body mutations	Urnov et al., 2005; Lombardo et al., 2007; Li et al., 2011; Genovese et al., 2014; Voit et al., 2014; Dever et al., 2016; Hubbard et al., 2016; Schiroli et al., 2017; Sweeney et al., 2017; Kuo et al., 2018; Wang et al., 2019, 2020a; Rai et al., 2020
B	Superactive promoters (ALB, HBA)	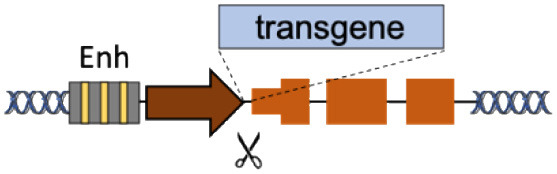	Accommodates different transgenes Supraphysiological expression Few integrations required	Partial gene disruption Limited to non-cell autonomous disorders Extensive validation required	De Ravin et al., 2016; Diez et al., 2017; Stephens et al., 2018, 2019; Gomez-Ospina et al., 2019; Scharenberg et al., 2020
C	Tolerant to integration (AAVS1, CCR5, Rosa26)	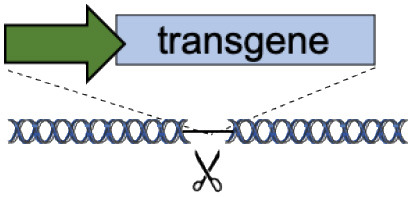	Accommodates different transgenes	Artificial promoters required Variable expression	De Ravin et al., 2016; Diez et al., 2017; Stephens et al., 2018, 2019; Gomez-Ospina et al., 2019; Scharenberg et al., 2020
D	Chromatin domains (NAD)	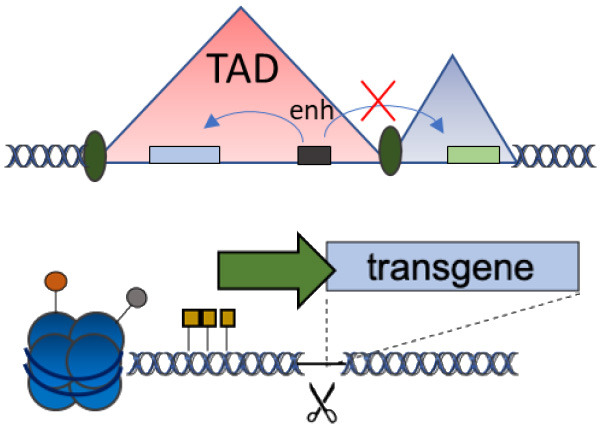	Fine gene regulation Far from oncogenic genes	No proof-of-principle in clinically relevant models	Schenkwein et al., 2020
E	Disease-modifier genes (CCR5, HBA)	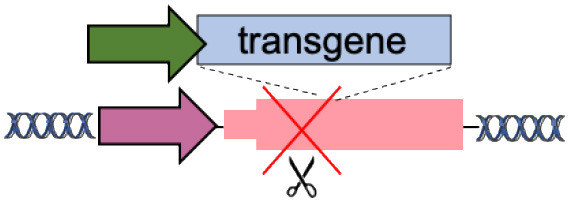	Improve therapeutic effect Lower therapeutic threshold	Extensive validation required Limited to well-known diseases	Voit et al., 2013; Wiebking et al., 2018
F	Specificity Exchange (TCR, BCR)	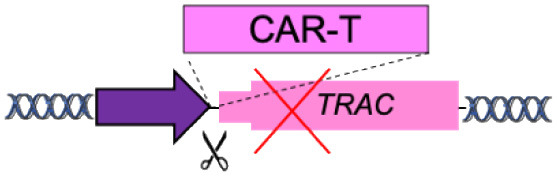	Improved CAR expression and potency	Off-targets Translocations risk (for multiple edits)	Eyquem et al., 2017; MacLeod et al., 2017; Greiner et al., 2019; Hartweger et al., 2019; Moffett et al., 2019; Voss et al., 2019

*Scissors: nuclease. Solid arrows indicate promoters, solid ovals indicate histone modifications, and solid squares indicate DNA modifications. Enh, enhancers; TAD, topologically associating, d, domain*.

The first proof of concept was obtained using ZFN on primary T cells *ex vivo* to replace interleukin-2 receptor subunit gamma (*IL2RG)*, whose mutational inactivation causes X-linked severe combined immunodeficiency (X-SCID) (Urnov et al., 2005; Lombardo et al., 2007). X-SCID represents an ideal model for testing this approach, as correction of only a small fraction of treated cells, given their strong growth advantage, should allow expansion and restoration of T cell function *in vivo*.

However, for effective clinical translation, targeted gene replacement should be performed in hematopoietic stem cells (HSC), the life-long source of all the different blood progenitors. Genovese via ZFN (Genovese et al., 2014) and Schiroli via CRISPR/Cas9 (Schiroli et al., 2017) were the first to report successful integration of a functional copy of *IL2RG* gene downstream its endogenous promoter in HSC, with the idea of restoring the endogenous lineage specificity and expression level of *IL2RG* without the risk of insertional mutagenesis (Hacein-Bey-Abina et al., 2003, 2008). Following this example, additional strategies have been developed for many blood diseases, including thalassemia (Voit et al., 2014; Dever et al., 2016), chronic granulomatous disease (De Ravin et al., 2017; Sweeney et al., 2017), hyper-immunoglobulin (Ig) M syndrome (Hubbard et al., 2016; Kuo et al., 2018), and Wiskott–Aldrich Syndrome (Rai et al., 2020).

Beside HSC and terminally differentiated blood cells, like B and T cells (Wang et al., 2016; Hung et al., 2018), AAV and nucleases have been the preferred method to achieve targeted transgene integration in many tissues *in vivo* (Suzuki et al., 2019; Kohama et al., 2020; Nishiguchi et al., 2020), especially the liver.

Li et al. were the first to demonstrate targeted gene correction *in vivo* by delivering ZFN and a partial *F9* (coagulation factor IX, FIX) cDNA cassette with AAV8 to the liver of a humanized mouse model of hemophilia B (Li et al., 2011). While correction was performed in newborn mice, FIX expression was maintained in adults and even persisted after partial hepatectomy, demonstrating stable genomic integration. This approach was later replicated using CRISPR/Cas9 to integrate a hyperactive FIX variant in the mouse *F9* locus (Wang et al., 2019).

Targeted gene replacement can also be combined with classical gene therapy to improve therapeutic outcome. In a neonatal mouse model of ornithine transcarbamylase (OTC) deficiency, an AAV carrying a liver-specific promoter and a human OTC transgene was integrated via CRISPR/Cas9 in the murine OTC locus (Wang et al., 2020a). Prompt, short-term expression from episomal AAV protected newborn mice from fatal hyperammonemia crisis, whereas its genomic integration allowed long-term disease correction.

Although targeting transgenes to their genomic loci is an effective therapeutic approach, it requires the development of countless gene-tailored editing strategies. Moreover, it can be difficult to reach and correct a number of cells that is sufficient to achieve a therapeutic benefit. Finally, its efficacy is limited in the presence of deletions/inversions that affect large portions of the locus or when regulatory elements controlling gene expression are mutated.

### Over/Expression by Superactive Promoters

Although gene-editing technologies are evolving at a fast pace, it can be challenging to correct enough cells to reach a clinical benefit even using high doses of nuclease and donor DNA, which increase chances of off-target genomic cleavage, immune responses, and donor random integration. An alternative strategy consists in “hijacking” strong endogenous promoters to overexpress therapeutic cassettes from few modified cells (Table 1B). An elegant example of this approach is the targeted integration of AAV-delivered transgenes under the control of the endogenous albumin promoter in the liver (Barzel et al., 2015; Sharma et al., 2015; Davidoff and Nathwani, 2016). Even with <1% of targeted integration events, the terrific transcriptional activity of this superactive promoter was sufficient to achieve 5–20% of FIX levels and correct bleeding in hemophilia B mice (Barzel et al., 2015). Until today, this strategy has been successfully applied in different preclinical models of hemophilia A and B (Barzel et al., 2015; Sharma et al., 2015; Chen et al., 2019; Conway et al., 2019; Zhang et al., 2019; Wang et al., 2020b) and metabolic disorders (Laoharawee et al., 2018; Conway et al., 2019; De Caneva et al., 2019; Ou et al., 2019). Importantly, this is also the first genome-editing strategy undergoing *in vivo* testing in humans to treat mucopolysaccharidosis I and II (NTC02702115, NTC03041324).

Although promising, this approach still presents some concerns. First, targeted integration can lower serum albumin levels (Zhang et al., 2019; Ou et al., 2020) and albumin mutations have been observed in human hepatocellular carcinoma (Cancer Genome Atlas Research Network, 2017; Rao et al., 2017). Second, long-term AAV-mediated expression of endonucleases can result in off-target editing and unwanted AAV insertions (Li et al., 2019; Breton et al., 2020; Wang et al., 2020c). Finally, pre-existing liver conditions and immune responses against AAV vectors used to deliver transgenes or nucleases severely limit the number of eligible patients (Boutin et al., 2010; Simhadri et al., 2018).

To avoid these issues, we have recently proposed to integrate therapeutic transgenes in the α-globin locus of HSC (Pavani et al., 2020). Similar to albumin targeting, the idea is to combine the strong transcriptional output of the α-globin promoter with the abundance of transgene-expressing erythroblasts to maximize protein production, reducing the number of integration events required to reach therapeutic levels. Moreover, differently from the liver, autologous HSC can be recovered from patients and edited *ex vivo* before re-administration, thus circumventing immunological issues. Additional experiments in preclinical disease models will elucidate the therapeutic potential of this novel HSC platform for treating genetic diseases.

Following these examples, additional endogenous promoters with specific expression levels/patterns can be exploited for transgene expression. Although promoter hijacking has many advantages over other approaches, it is important to functionally validate the dispensability of the disrupted gene, as nuclease-mediated targeting can result in bi-allelic gene knock out, or to consider safer editing alternatives (e.g., nicking endonucleases Ran et al., 2013).

## Safe Genomic Harbors

### Tolerant to the Integration of an Expression Cassette

Genomic safe harbors are intragenic or intergenic regions of the human genome that enable stable expression of integrated transgenes without negatively affecting the host cell (Sadelain et al., 2011). Targeting expression cassettes to these loci is an efficient way to develop a “one-fits-all” platform to express different therapeutic transgenes using the same nuclease(s), therefore optimizing efficiency and improving safety.

By far, the most widely targeted genomic loci are AAVS1, CCR5, and Rosa26 (Table 1C).

The *AAVS1 locus* (chromosome 19 q13.42) was historically identified as the preferential integration site of wild-type AAV in human cell lines (Kotin et al., 1992). It encodes the PPP1R12C gene, a subunit of myosin phosphatase whose functions are not fully elucidated (Surks et al., 2003), but probably redundant (Smith et al., 2008). Stable and corrective editing of patients' HSC at this locus has been obtained by integrating a transgene cassette with (Fanconi anemia (Diez et al., 2017)) or without an exogenous promoter (X-CGD (De Ravin et al., 2016)). It is worth noting that the AAVS1 locus is an extremely gene-rich region and, although the presence of an insulator in the promoter of PPP1R12C could shield the genome from the action of the inserted promoter/enhancer (Ogata et al., 2003; Li et al., 2009), it requires a carefully designed transgene expression cassette to avoid transcriptional perturbation of neighboring genes (Lombardo et al., 2011). Moreover, several studies showed that variable expression and promoter silencing can occur at this site in different cell types (Lamartina et al., 2000; Smith et al., 2008; Ordovas et al., 2018; Bhagwan et al., 2019; Klatt et al., 2020), thus potentially limiting transgene expression.

The *CCR5* gene (chromosome 3 p21.31) encodes for the main HIV co-receptor. Since a bi-allelic null mutation of this receptor (CCR5Δ32) confers HIV-1 resistance and is not associated with any major pathology (Hutter et al., 2009), this locus was first targeted/disrupted with nucleases in T cells and HSC to provide protection against AIDS ((Perez et al., 2008; Yu et al., 2020), NCT00842634, NCT02500849, and NCT03164135) and later exploited for targeted gene addition. Therapeutic transgenes involved in lysosomal storage disorders were inserted in the *CCR5* gene of human HSC, under the control of exogenous ubiquitous or tissue-specific promoters. Upon transplantation, edited HSC engrafted, differentiated, and corrected the pathological phenotype in mouse models of MPS I (Gomez-Ospina et al., 2019) and Gaucher (Scharenberg et al., 2020). Although promising, the safety of this approach needs to be further validated, as CCR5 deficiency can result in increased susceptibility to West Nile (Lim et al., 2006; Cahill et al., 2018), influenza (Falcon et al., 2015), and Japanese encephalitis viruses (Larena et al., 2012).

The *Rosa26 locus* (chromosome 3 p25.31) was serendipitously discovered in mice as a reliable site to integrate DNA cassettes for transgenesis (Zambrowicz et al., 1997). This locus was then successfully targeted *in vivo* with CRISPR/Cas9 to knock-in human alpha-1-antitrypsin or FIX in mouse liver (Stephens et al., 2018, 2019). The human homolog was identified on chromosome 3 (position 3p25.3) (Irion et al., 2007); however, the efficacy and safety of this site for targeted integration is still undetermined.

While genomic safe harbors could represent a universal platform for gene targeting and thus expedite clinical development, so far no site of the human genome has been fully validated. The described loci may be acceptable for research applications, but clinical translation will require extensive validation as they localize in gene-dense areas and in proximity of cancer-related genes.

### Chromatin Domains With Specific Expression Patterns

The genomic location of transgene integration can change its transcription up to 1,000-fold, according to some well-studied aspects of large-scale domain organization of chromatin (Akhtar et al., 2013; Brueckner et al., 2016; Corrales et al., 2017). Recent evidence for targeting 3D chromatin domains comes from the work of Schenkwein et al. showing that in primary human T cells genomic regions distant from one another linearly, but near in the three-dimensional genome, became jointly affected when site-specific transgene integration was performed (Schenkwein et al., 2020). In this work, transgenes were targeted to nucleolar-associated domains (NAD), which are distant from protein-encoding genes with oncogenic potential and thus represent safe genomic loci for inserting therapeutic transgenes.

The increasing knowledge of chromatin functions and dynamics (Moore et al., 2020) might soon allow us to select integration sites to obtain a certain transcriptional activity and cell/tissue/developmental specificity, as predicted by the presence/absence of certain histone marks (Talbert et al., 2019), DNA methylation, transcriptional factor binding sites, nuclear lamina interaction (Amendola and van Steensel, 2014), chromatin accessibility, and topology (Zheng and Xie, 2019; Zhang et al., 2020) (Table 1D). We can easily envision that the combination of selected chromatin locations and expression cassettes will allow fine-tuning of therapeutic transgene expression to unprecedented levels.

## Disease-Modifier Genes

### Inactivation of Pathogen Receptors

A disease-modifier gene alters the expression of another gene involved in a genetic/infectious disorder, therefore changing the penetrance, dominance, and severity of the disease itself (Genin et al., 2008). Novel genome-editing strategies can combine transgene expression with modulation of disease-modifier genes to improve therapeutic outcomes and provide cells with novel functions (Table 1E). Voit et al. were the first to describe the use of ZFN to integrate transgenes encoding for HIV restriction factors into the HIV co-receptor gene CCR5 (Voit et al., 2013). With this strategy, treated T cells were resistant to HIV infection thanks to the concomitant expression of protective transgenes and knockout of CCR5 (disease-modifier).

### Restoring Balance in Disease Pathways

A second example of this approach involves β-thalassemias, a group of blood disorders caused by mutations in the β-globin gene. β-globin associates with α-globin to form adult hemoglobin (HbA, α2β2) and, when β-globin chains are absent or limiting, free α-globin precipitates causing hemolysis and ineffective erythropoiesis. Reduction of α-globin has been shown to ameliorate the β-thalassemia phenotype (Mettananda et al., 2015); hence, we and others have proposed to target the integration of a β-globin transgene into the α-globin site (disease-modifier) of HSC to simultaneously express the therapeutic gene while reducing α-globin production in differentiated erythroblasts (Table 1E) (Pavani et al.; Cromer et al.; Molecular Therapy Vol 27 No 4S1, April 2019). The full potential of this combination therapy for these and other genetic diseases will be more clear in the future (Hightower and Alexander, 2018; Rahit and Tarailo-Graovac, 2020).

While the possibility of combining gene replacement and endogenous gene regulation could attain unparalleled additive or synergic therapeutic effects, it is limited to the treatment of diseases for which a deep knowledge of the underlying molecular mechanism is available, and it requires careful examination.

### Providing Novel Functions

Targeted integration can also provide cells with novel functions, such as a “safety-switch” for cell therapy applications. Transgene integration can be directed to inactivate an essential metabolic enzyme, the uridine monophosphate synthetase, which makes T cells dependent on supplemented uridine for their growth and survival (Wiebking et al., 2018). This approach could help therapies based on chimeric antigen receptor T cells by introducing a metabolic control of their proliferation and persistence. Further experiments are required to evaluate the clinical readiness of the approach.

## Specificity Exchange

A special case of gene targeting is represented by the “specificity exchange” (Table 1F). Chimeric antigen receptors (CARs) are synthetic receptors that redirect and reprogram T cells to recognize specific antigens for tumor rejection (June and Sadelain, 2018). Initially, CARs were introduced in T cells using retroviral and lentiviral vectors (gene addition), with the risk of insertional mutagenesis. In addition, these CAR-T cells had two antigen specificities, the engineered one and the physiological one encoded by the endogenous αβ T cell receptor (TCR) chains, which may induce graft-vs-host disease when allogenic T cells are used (Torikai et al., 2012).

New CAR-T cells are generated by targeting the integration of the CAR transgene under the transcriptional control of TCR α-chain gene promoter to simultaneously achieve physiological expression of CAR and disruption of the endogenous TCR, thus maintaining only CAR antigen specificity (specificity exchange) (Eyquem et al., 2017; MacLeod et al., 2017). Overall, this strategy allows uniform CAR expression in human T cells and enhances T cell potency, outperforming conventional CAR-T cells.

A similar strategy has also been described to integrate and express a sequence encoding for a defined monoclonal antibody (Ab) of interest under the control of the heavy or light immunoglobulin chain promoter to reprogram B cells to secrete broadly neutralizing Ab against pathogens, for which no protective Ab has been isolated (Greiner et al., 2019; Hartweger et al., 2019; Moffett et al., 2019; Voss et al., 2019).

## Conclusions

Over the past decades, gene therapy for blood disorders has mainly focused on the optimization of transgenes and synthetic promoters to improve expression and achieve therapeutic effects using gene replacement. However, this strategy is associated with the risk of insertional mutagenesis (LV) and episomal vector loss (AAV). The advent of the first generation of DNA endonucleases allowed the integration of transgenes in few selected genomic loci, mainly to achieve stable expression while minimizing insertional mutagenesis risk. Now, thanks to easily programmable nucleases such as CRISPR/Cas9, we have dramatically expanded our integration options and can creatively exploit different genomic locations to finely tune transgene expression or modulate disease-modifier genes to improve gene therapy outcomes.

A common strategy to target transgene integration combines nucleases with a donor DNA template (generally AAV) and leverages the homologous recombination pathway. However, before clinical translation, strict functional validation will be necessary to reduce potential adverse events associated with each individual component of this system. In particular, nucleases can induce potential off-targets (Kleinstiver et al., 2016; Carroll, 2019) and chromosomal alterations induced by on-target cleavage (Adikusuma et al., 2018; Kosicki et al., 2018; Cullot et al., 2019; Ledford, 2020); nucleases and AAV activate p53 response and trigger cell cycle arrest (Schwartz et al., 2007; Haapaniemi et al., 2018; Ihry et al., 2018); donor DNA integration can occur by different DNA repair mechanisms with outcomes sometimes difficult to predict (Canaj et al., 2019; Hanlon et al., 2019; Nelson et al., 2019); the target site needs to be functionally validated for safety and disposability (Papapetrou and Schambach, 2016).

Additional studies and further optimization of existing editing technologies will remove these hurdles and allow a broad clinical application of the described strategies to treat both monogenic and multifactorial blood diseases.

## Author Contributions

GP and MA wrote the manuscript. All authors contributed to the article and approved the submitted version.

## Conflict of Interest

The authors declare that the research was conducted in the absence of any commercial or financial relationships that could be construed as a potential conflict of interest.
