# Corrigendum: Targeted Gene Delivery: Where to Land

**DOI:** 10.3389/fgeed.2021.682171

**Published:** 2021-05-17

**Authors:** Giulia Pavani, Mario Amendola

**Affiliations:** INTEGRARE, UMR_S951, Genethon, Inserm, Univ Evry, Univ Paris-Saclay, Evry, France

**Keywords:** genome editing, gene therapy, nuclease, CRISPR, targeted integration (TI), knock-in, safe harbor, homologous recombination (HR)

In the original article, there was a mistake in Table 1 as published. The references indicated in row **B** are wrong. The corrected Table 1 appears in the attached below.

**Table 1:** **(A–F)** The advantages and disadvantages of different integration strategies.

	**Integration strategies**		**Advantages**	**Disadvantages**	**References**
A	Endogenous locus	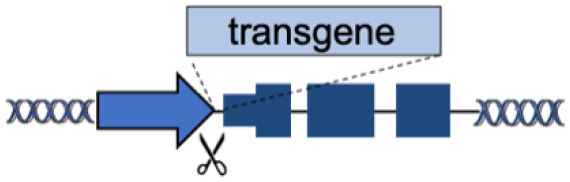	Physiological transgene expression Corrects multiple mutations	Gene-specific strategy Limited to gene body mutations	Urnov et al., 2005; Lombardo et al., 2007; Li et al., 2011; Genovese et al., 2014; Voit et al., 2014; Dever et al., 2016; Hubbard et al., 2016; Schiroli et al., 2017; Sweeney et al., 2017; Kuo et al., 2018; Wang et al., 2019; Rai et al., 2020; Wang L. et al., 2020
B	Superactive promoters (ALB, HBA)	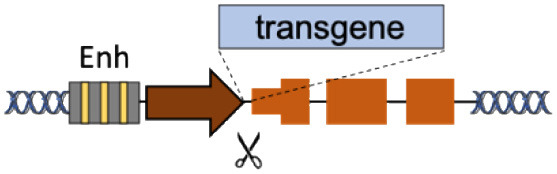	Accommodates different transgenes Supraphysiological expression Few integrations required	Partial gene disruption Limited to non-cell autonomous disorders Extensive validation required	Barzel et al., 2015; Sharma et al., 2015; Davidoff and Nathwani, 2016; Laoharawee et al., 2018; Chen et al., 2019; Conway et al., 2019; De Caneva et al., 2019; Ou et al., 2019, 2020; Zhang et al., 2019; Wang Q. et al., 2020
C	Tolerant to integration (AAVS1, CCR5, Rosa26)	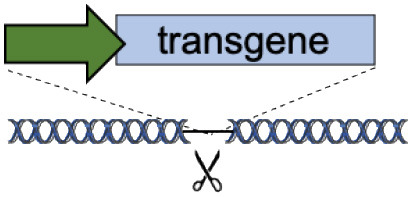	Accommodates different transgenes	Artificial promoters required Variable expression	De Ravin et al., 2016; Diez et al., 2017; Stephens et al., 2018, 2019; Gomez-Ospina et al., 2019; Scharenberg et al., 2020
D	Chromatin domains (NAD)	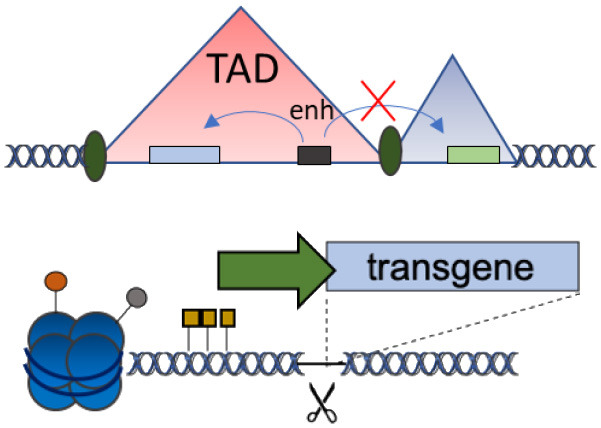	Fine gene regulation Far from oncogenic genes	No proof-of-principle in clinically relevant models	Schenkwein et al., 2020
E	Disease-modifier genes (CCR5, HBA)	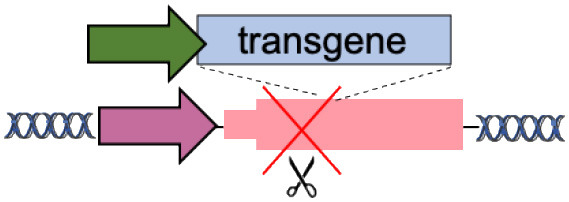	Improve therapeutic effect Lower therapeutic threshold	Extensive validation required Limited to well-known diseases	Voit et al., 2013; Wiebking et al., 2018
F	Specificity Exchange (TCR, BCR)	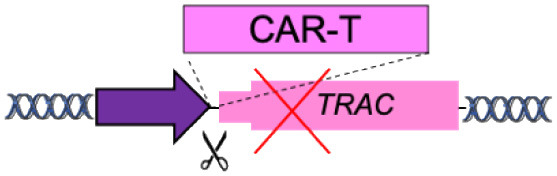	Improved CAR expression and potency	Off-targets Translocations risk (for multiple edits)	Eyquem et al., 2017; MacLeod et al., 2017; Greiner et al., 2019; Hartweger et al., 2019; Moffett et al., 2019; Voss et al., 2019

*Scissors: nuclease; Solid arrows: promoters; Enh, enhancers; TAD, topologically associating; d, domain; Solid ovals: histone modifications; Solid squares: DNA modifications*.

The authors apologize for this error and state that this does not change the scientific conclusions of the article in any way. The original article has been updated.
